# A Comparison of the Apical Extrusion of Debris during the Preparation of Root Canal with Medin, RaCe, and ProTaper Rotary Systems

**DOI:** 10.30476/DENTJODS.2020.84776.1100

**Published:** 2021-09

**Authors:** Mohammadreza Nabavizadeh, Mohammad Mehdi Shokouhi, Mojgan Kheirandish, Safoora Sahebi, Nooshin Sadatshojaee, Abbas Abbaszadegan

**Affiliations:** 1 Oral and Dental Disease Research Center, Dept. of Endodontics, School of Dentistry, Shiraz University of Medical Sciences, Shiraz, Iran; 2 Dept. of Endodontics, School of Dentistry, Shiraz University of Medical Sciences, Shiraz, Iran; 3 Dental Student, School of Dentistry, Shiraz University of Medical Sciences, Shiraz, Iran

**Keywords:** Apical extrusion, Debris, Endodontic treatment, Rotary file

## Abstract

**Statement of the Problem:**

The extrusion of intracanal debris is one of the challenging problems related to almost all root canal preparation systems, which may cause flare-ups and impairment in the healing process.

**Purpose:**

This study was conducted to evaluate the amount of apically-extruded debris during root canal preparation using Medin (MEDIN Co., Czech Republic) rotary system compared with two common rotary systems, including ProTaper (Dentsply Maillefer., Switzerland) and RaCe (FKG Dentaire, Switzerland).

**Materials and Method:**

In this *in vitro* study, Sixty mandibular premolars with single canal were randomly assigned to three groups (n=20). The root canals were prepared with Medin, ProTaper, and RaCe rotary instruments based on their manufacturers’ instructions. The debris were collected into pre-weighted Eppendorf tubes. The weight of the extruded debris was calculated by subtracting the pretreatment weight of the vials. Data were analyzed using the Kruskal-Wallis test at a 5% significance level.

**Results:**

Medin instrument caused significantly less debris extrusion in comparison with ProTaper and RaCe (*p*< 0.05). The differences between the ProTaper and RaCe rotary systems were not statistically significant (*p*= 0.752).

**Conclusion:**

Within the limitations of this *in vitro* study, Medin rotary system produced less apical extrusion than ProTaper and RaCe.

## Introduction

The importance of mechanical root canal preparation has persuaded manufacturers to introduce endodontic rotary files with different design features, kinematics, and advantages [ 1 ]. One of the frustrating problems related to almost all root canal preparation systems, which may cause flare-ups and affect the healing process is the debris extrusion [ 2 - 3 ]. 

Medin (MEDIN Co., Czech Republic) rotary files with inactive tips and a three-bladed profile are designed to shape curved canals using the crown-down technique. The manufacturers claim that the resistance of files to cyclic fatigue increases by special heat treatment processing. Several Studies have compared this rotary system with popular rotary systems in terms of shaping abilities. Bidar *et al*. [ 4 ] microscopically compared the cleaning efficiency of this rotary system with RaCe and Mtwo instruments and did not find any differences between the groups. In another study, Moradi *et al*. [ 5 ] compared the dentin removal and centering ability of these three rotary file systems in curved canals and found that Mtwo is more conservative for root canal preparation. Talati *et al*. [ 6 ] also found the superiority of Mtwo over RaCe and Medin rotary systems regarding the avoidance of apical transportation. Several studies have considered the apical debris extrusion of popular rotary systems such as ProTaper (Dentsply Maillefer, Switzerland) and RaCe (FKG Dentaire, La Chaux-de-Fonds, Switzerland) rotary systems [ 7 - 10 ]. 

Given the lack of published studies to date on Medin rotary NiTi instruments concerning apical extrusion, the present study was performed to evaluate the amount of apical-debris extrusion during root canal preparation using this rotary system compared with RaCe and ProTaper rotary systems.

## Materials and Method

The Ethics Committee of Shiraz University of Medical Sciences approved this study (IR.SUMS.REC. 1394. S970). A total of 60 mandibular premolar teeth that had been recently extracted for periodontal and orthodontic reasons were evaluated at 32x magnification (Bestscope, BS-3060C) and radiographed in both buccolingual and mesiodistal directions. The inclusion criteria for samples were considered as being a straight root with mature apex with a single apical foramen not laterally opened, without caries, resorptive area, calcification, or fractures and without any previous restorative or endodontic procedures. After removing the calculi and periodontal remnant from the root surface, the access cavity was prepared and the canal length was measured by insertion of a #15 K-file (Mani, Takanezawa, Japan) in the canal until the tip end was seen in the apical foramen. The working length was recorded as 1 mm short of this length. The length of all the roots were standardized by removing the excess coronal reference points perpendicular to the long axis. The extrusion debris test was performed based on the method proposed by Montgomery and Meyers with some modifications [ 11 ]. The samples were randomly sorted into three groups (n=20) and marked. The root surfaces were covered with a Teflon band, except for 1 mm in the apical part. The teeth were then placed in holes created in the center of the cap of the Eppendorf tubes and fixed at the cementoenamel junction. Sixty Eppendorf tubes without caps were weighted three times to 10-4g precision using a digital scale (GT300, A&D Company, Japan). The mean value of these consecutive measurements was recorded for each tube. The apical part of the roots was positioned inside the Eppendorf tubes and sealed by cyanoacrylate adhesive. In order to balance the air pressure inside and outside of the tube, a 25-gauge needle was inserted and secured in caps by the side of each tooth. The whole assembly was secured in a brown glass to prevent any movement during cleaning and shaping procedures.

### Root canal preparation

All the root canal preparations were done by one expert operator using new files. After every three pecking movements, the root canals were irrigated with 2 ml of distilled water. The debris on the files was wiped off by gauze soaked in alcohol. A #10 K file was used for the recapitulation and the canal was again irrigated with 2 ml of distilled water. A total of 20 ml of distilled water irrigation was performed and completed for each sample until preparation using a 30-Gauge needle at the flow rate of approximately 4 ml/min.

The final rinse was performed in all the groups by placing the needle 2 ml short of the working length. In all the groups, the files were applied with an Endo Mate DT (NSK, JAPAN) endodontic motor according to the speed and torque value suggested by the manufacturers of the rotary systems.

### Group 1

ProTaper files were used as follows: SX at two-thirds of the working length followed by S1 and S2 at 1mm short of the working length and instrument of F1 (20/0.07), F2 (25/0.08), F3 (30/0.09) at the working length

### Group 2

RaCe instruments were used in a crown-down manner with a gentle in and out motion. File sequences used were: #25, 0.06 taper until half of the working length followed by #25, 0.04 taper used between half and 2/3 ^rd^ of working length and instruments of #20, 0.02 taper, #25,0.02 taper, and #30, 0.02 taper simultaneously used to their working length 

### Group 3

Medin (AS, Czech Republic) files were used as follows: #25, 0.07 taper until half of the working length followed by #10, 0.04 and #15, 0.05 taper at 1mm short of working length and instruments of #20, 0.06 taper, #25,0.06 taper, and #30, 0.06 taper simultaneously used to their working length. After the root canal preparation, the teeth were detached from the Eppendorf tubes and rinsed with 1 ml of distilled water to detach the remnant of the debris and add them to the tube. Next, each tube was weighted consecutively three times. The weight of the apparatus without tooth was subtracted from the weight of the tubes with dry debris. The tubes were kept for five days at 68 °C in an incubator to remove the water content. The measurements were repeated three times with an accuracy of 10^-4^ g and their mean value was recorded.

**Figure 1:**
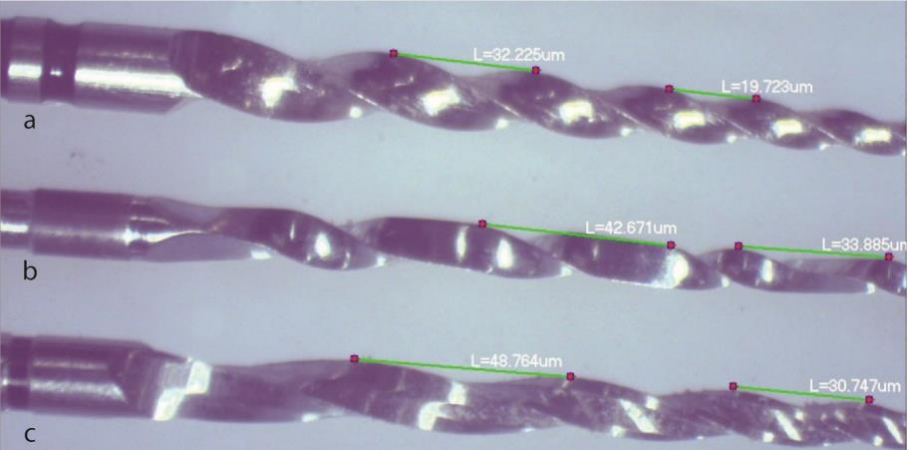
a: Medin, b: RaCe and c: ProTaperSPSS 21.0 software (IBM Corp, Armonk, NY) was used to perform the statistical analysis. An independent t-test was employed to compare debris extrusion between the groups.

In order to evaluate the role of pitch length in debris extrusion, the #30 file of each system was photographed by microscope (Bestscope, BS-306C) at ×8 magnification. The pitch length was then recorded using image scope program (the Leica Biosystems group / USA) (Figure 1).

## Results

Table 1 shows the mean and standard deviation of the amount of apically extruded debris for each group. According to Table 1, Medin rotary system extruded significantly less debris from the apex than ProTaper and RaCe (*p*= 0.001 and *p*= 0.007 respectively). There were no significant differences between debris extrusion by the ProTaper and RaCe systems (*p*= 0.752). 

**Table1:** Amount of apically extruded debris (in grams) after the use of the different instruments

Group	N	Mean	Std.Deviation
ProTaper	20	0.0105	0.0038
RaCe	20	0.0096	0.0046
Medin	20	0.0056	0.0032

## Discussion

Several laboratory studies have been conducted to evaluate the cleaning efficiency, centering ability and amount of apical transportation of Medin rotary systems [ 4 - 6 ]. To the researchers’ knowledge, this study is the first to compare the apical extrusion of the debris of this rotary system with two commonly used rotary systems, including RaCe and ProTaper. The results of this study revealed that all the tested rotary systems produced apically extruded debris *in vitro*.

In this study, the lowest amount of debris extrusion was observed with Medin rotary NiTi instruments in comparison with RaCe and ProTaper rotary systems. The present findings are in accordance with the results of several studies that have reported ProTaper systems to extrude the largest amount of debris among other tested systems [ 10 , 12 - 13 ]. Apart from the S files, the ProTaper system prepared the apical end of the canal in the early stage of root canal preparation, which may explain the large amount of apical extrusion in this file system. In two studies performed by Soi *et al*. [ 14 ] and Altundasar *et al*. [ 15 ], ProTaper exhibited more debris extrusion than RaCe rotary systems. The non-convex, triangular, cross-sectional design of RaCe systems, their smaller core diameter and short, twisted cutting edges alternating with straight edges were taken as the reason for these findings. In ProTaper rotary systems, the coronal flow of irrigant is limited by the sudden flaring of ProTaper files. The term Pitch has been applied to the distance between the edges of two cutting blades measured along the working part of an instrument [ 16 - 17 ]. The length of pitch can change the mechanical properties of rotary files; a study performed by Burklein *et al*. [ 17 ] showed that the files with shorter pitch had greater contact area with root canal walls and thereby caused more torsional stress during instrumentation. In another study conducted by Elmsallati *et al*. [ 18 ] on the effect of pitch length on the extrusion of debris, a short pitch design resulted in significantly less amounts of debris extrusion compared to long ones. The shorter pitch length in the Medin rotary system may explain the less debris extrusion in this group compared with RaCe and ProTaper.

The amount of debris extrusion also depends on instrument type, size, and working length [ 19 - 20 ]. In this study, all the samples were prepared 1mm short of the apical foramen to the ISO size of 30; also, teeth with similar type, canal size, and curvature were selected to that only the design of the rotary systems could determine the amount of debris extrusion. 

This study employed Myers and Montgomery model [ 11 ] to evaluate the amount of apical extrusion. This system is not the same as the pulpal and periapical tissue and their resistance to the extrusion of debris. Consequently, the results cannot be directly expected in clinical conditions. The use of materials that simulate the periapical tissue, such as agar, alginate, or floral foam, may underestimate the amount of debris extrusion by absorbing some debris. These media were therefore not used in this study [ 21 - 22 ]. 

To prevent the probability of crystallization of common irrigants, such as sodium hypochlorite in the collection tube, distilled water was used as irrigant during the preparation [ 22 ]. 

The present findings are restricted to teeth with mature apices and cannot be extrapolated to teeth with open apices. There are some non-standardizable factors, such as dentin micro-hardness in human teeth models, which may influence the amount of apical extrusion [ 2 ]; however, considering possible adverse effects related to simulated acrylic blocks, such as the effect of heat on the hardness of resin material, made us use human teeth models in this study.

## Conclusion

Within the limitations of this *in vitro* study, the findings showed that, during canal preparation, Medin rotary systems produced less apical extrusion of debris compared to ProTaper and RaCe rotary systems.
